# Bridging the gap between enthusiasm and competency: a national survey of knowledge, attitudes, and readiness for dental stem cell therapies among Saudi dental practitioners

**DOI:** 10.3389/froh.2026.1801062

**Published:** 2026-04-10

**Authors:** Mohammad Zakaria Nassani, Anas Alsalhani, Faisal Mehsen Alali, Nasser Raqe Alqhtani, Abdullah Saad Alqahtani, Abdullah bin nabhan, Yousef Alkhaibari, Abdullah Almansour, Rawda Omar Alghabban, Mohammed Alkhathlan, Ahmad Almughirah, Fahad Alsultan, Ali AlRafedah, Bassel Tarakji

**Affiliations:** 1 Department of Restorative and Prosthetic Dental Sciences, College of Dentistry, Dar Al Uloom University, Riyadh, Saudi Arabia; 2 Department of Removable Prosthodontics, Faculty of Dentistry, University of Aleppo, Aleppo, Syria; 3 Department of Dentistry, Vision Colleges, Riyadh, Saudi Arabia; 4 Department of Histology and Pathology, Faculty of Dentistry, University of Hama, Hama, Syria; 5 Department of Oral Maxillofacial Surgery and Diagnostic Sciences, College of Dentistry, Prince Sattam Bin Abdulaziz University, Al-Kharj, Saudi Arabia; 6 Department of Preventive Dental Sciences, College of Dentistry, Prince Sattam Bin Abdulaziz University, Al-Kharj, Saudi Arabia; 7 Department of Oral and Maxillofacial and Diagnostic Science, College of Dentistry, Prince Sattam Bin Abdulaziz University, Al-Kharj, Saudi Arabia; 8 Department of Pediatric Dental Sciences, College of Dentistry, Prince Sattam Bin Abdulaziz University, Al-Kharj, Saudi Arabia; 9 Prince Sattam Bin Abdulaziz University, College of Dentistry, Al-Kharj, Saudi Arabia; 10 Department of Histology and Pathology, Faculty of Dentistry, University of Aleppo, Aleppo, Syria

**Keywords:** dental stem cells (DSCs), stem cell therapy, regenerative dentistry, dental practitioners, cross-sectional survey, dental pulp stem cells (DPSCs), Saudi Arabia, knowledge attitudes and practices (KAP)

## Abstract

**Background:**

Regenerative dentistry, driven by dental stem cells (DSC), offers biologically based approaches for tissue repair and regeneration. Despite increasing scientific advances, clinical translation depends largely on dental practitioners' knowledge, attitudes, and readiness to adopt these therapies. In Saudi Arabia, where biomedical innovation is emphasized under Vision 2030, the integration of DSC-based applications into routine dental practice remains insufficiently understood.

**Objective:**

This study assessed the level of knowledge, attitudes, and clinical practice intentions (KAP) regarding dental stem cell applications among dental practitioners in Saudi Arabia and identified key demographic and professional predictors.

**Methods:**

A nationwide, cross-sectional, online survey was conducted between November 2024 and August 2025 using a validated questionnaire. A total of 493 fully completed responses from actively practicing dentists were analyzed. Descriptive statistics summarized KAP outcomes, while chi-square tests and multivariate binary logistic regression were used to examine associations and independent predictors of good knowledge, positive attitude, and favourable clinical practice intention.

**Results:**

A pronounced knowledge–practice gap was identified. Although 83.3% of participants demonstrated a positive attitude toward DSC-based regenerative therapies, only 16.7% exhibited good knowledge and 50.1% reported good clinical practice intention. Prior education on stem cells was the strongest independent predictor of both good knowledge (OR=2.13, 95% CI: 1.43–3.19; *p* < 0.001) and positive attitude OR=2.10, 95% CI: 1.31–3.36; *p* = 0.002). Specialist qualification (OR=2.03, 95% CI: 1.23–3.36; *p* = 0.006) and mid-career status (31–50 years) (OR=2.03, 95% CI: 1.27–3.24; *p* = 0.003) were also significant predictors of a positive attitude, while no factors independently predicted clinical practice intention.

**Conclusion:**

Saudi dentists show strong enthusiasm for regenerative dentistry but limited knowledge and clinical readiness. These findings highlight the need for structured curricular integration, targeted continuing professional development, and supportive regulatory frameworks to bridge the gap between theoretical interest and the safe, effective clinical application of dental stem cell–based therapies in Saudi Arabia.

## 1 Introduction

Regenerative dentistry represents a transformative paradigm shift from conventional restorative approaches toward biologically driven tissue repair and regeneration (1, 2). At the core of this innovative field are stem cells, characterized by their self-renewal capability and potential to differentiate into specialized cell types essential for the regeneration of dental and craniofacial tissues. Among the various stem cell populations, dental stem cells (DSC)—including those derived from the dental pulp, apical papilla, periodontal ligament, follicle, and exfoliated deciduous teeth—have emerged as a promising, accessible, and minimally invasive source for regenerative therapies (3–5).

**Graphical Abstract:**
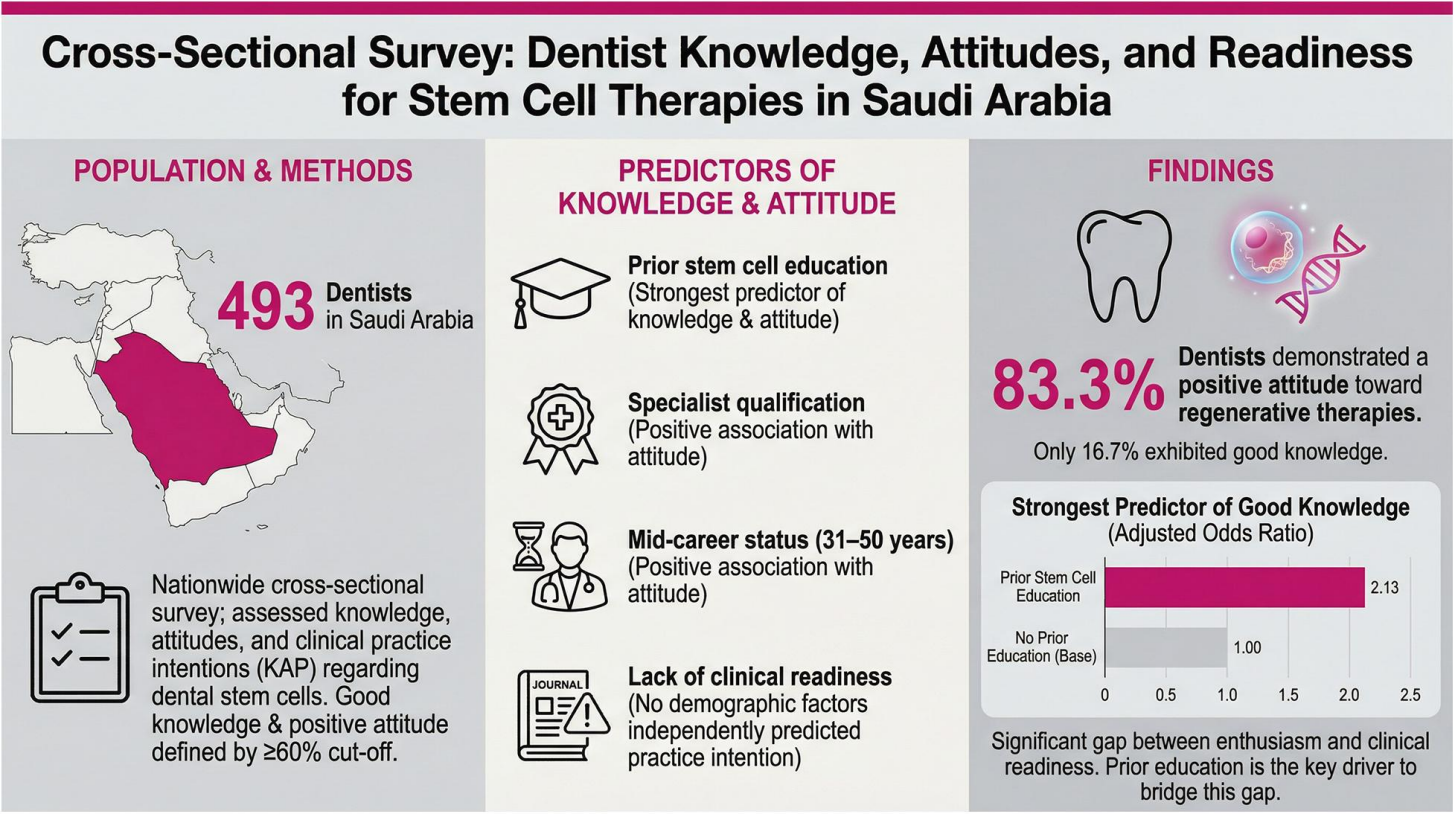


Over the last two decades, the development of regenerative endodontics and tissue engineering has gained remarkable attention as a viable alternative to traditional treatment modalities such as root canal therapy and apexification. Unlike conventional restorative approaches that aim merely to repair or replace damaged tissues, regenerative dentistry seeks to restore vitality, structure, and function to the pulp-dentin complex through biologically active materials and cellular mechanisms. Recent reviews have highlighted the integration of stem cells, scaffolds, and bioactive signalling molecules as key components driving pulp regeneration and clinical translation (6–8). However, the clinical translation of DSC-based therapies relies not only on scientific progress but also on the awareness, acceptance, and readiness of dental professionals to incorporate these advanced techniques into practice (9).

Despite growing evidence supporting dental stem cell (DSC) applications in pulp regeneration, root development, periodontal repair, and even whole-tooth engineering (10–12), their routine clinical use remains limited worldwide (13). Although dental stem cell (DSC)–based regenerative therapies have demonstrated considerable promise, their routine clinical use remains limited. Most DSC applications are currently in the preclinical research or early clinical trial phases, particularly in areas such as pulp–dentin complex regeneration, periodontal tissue repair, and craniofacial bone regeneration (6–10)**.** Experimental studies and early clinical investigations have reported encouraging outcomes, including continued root development, apical closure, dentin formation, and improved tissue vitality in immature teeth treated with regenerative endodontic procedures (10–12). Despite these promising results, widespread clinical implementation remains constrained by regulatory, technical, and infrastructural challenges, as well as the need for long-term clinical evidence regarding safety and efficacy (13).

This gap between research and practice is particularly evident in developing regions, where challenges related to education, clinical infrastructure, and regulatory frameworks persist. Understanding how practitioners perceive these innovations is therefore crucial for the successful integration of regenerative dentistry into daily clinical care.

In the Kingdom of Saudi Arabia, the field of dentistry has undergone significant advancement, supported by national efforts to modernize healthcare infrastructure and prioritize biomedical innovation in line with Vision 2030 (14). The government's investment in research institutions such as the King Abdullah International Medical Research Center and initiatives like the Saudi Stem Cell Donor Registry highlight a strong national commitment to promoting regenerative medicine. However, while stem cell science has gained considerable attention, its specific application in dental practice remains underexplored (15). Recent evidence from Saudi Arabia indicates that public awareness and understanding of stem cell–based therapies remain limited, with many individuals expressing uncertainty about their clinical applications, ethical aspects, and long-term safety and cost-effectiveness (16). This general lack of awareness likely extends to dental practice, where the integration of regenerative concepts is still emerging.

Educational exposure plays a central role in shaping practitioner attitudes toward new technologies. Dentists who actively participate in continuing professional development programs, scientific conferences, and regenerative dentistry courses are more likely to exhibit positive perceptions and confidence in applying such treatments (17). Nevertheless, knowledge gaps persist regarding stem cell sources, harvesting techniques, storage and banking procedures, clinical safety, and the regulatory governance required to ensure ethical and effective implementation (18). Addressing these gaps is essential to facilitate the safe and evidence-based integration of regenerative procedures into the mainstream dental healthcare system in Saudi Arabia.

Clinical conditions such as the management of necrotic immature teeth exemplify the growing need for regenerative alternatives to conventional apexification. Studies have shown that stem cell–based pulp regeneration can successfully stimulate root maturation, dentin formation, and apical closure, resulting in better long-term tooth preservation compared to traditional approaches using materials such as mineral trioxide aggregate (MTA) or calcium hydroxide (11, 19). These findings emphasize the critical need to bridge the gap between research and clinical application in regenerative dentistry.

Beyond dentistry, DSC exhibit remarkable multilineage differentiation capabilities, including neural, vascular, and osteogenic potential, thus extending their utility into systemic regenerative medicine (20). This broader therapeutic relevance reinforces the need for interdisciplinary collaboration among dentists, medical researchers, and policymakers to advance the application of regenerative medicine in Saudi Arabia (21).

Attitudinal and behavioural factors further influence the adoption of innovative treatments. Practitioners' perceptions regarding safety, clinical efficacy, ethical acceptability, cost, and patient demand often determine whether they are willing to recommend or adopt stem cell–based therapies in clinical practice (22). Research suggests that clinicians who perceive regenerative techniques as superior to implants or prosthetic alternatives are more likely to endorse their use (23). Therefore, assessing these perceptions provides valuable insights into potential barriers and facilitators of clinical implementation.

Given the global shift toward personalized and biologically guided treatment paradigms, it is imperative to understand Saudi dental practitioners' current levels of knowledge, attitudes, and readiness to engage with stem cell–based applications. This need aligns with Saudi Arabia's Vision 2030 objectives, which emphasize advancing biomedical innovation, strengthening health research capacity, and promoting translational education to bridge the gap between emerging science and clinical application (24).

This study aimed to evaluate the current level of knowledge, attitudes, and clinical practice intentions (KAP) regarding (DSC)–based regenerative therapies among dental practitioners in Saudi Arabia. Particular emphasis was placed on identifying demographic and professional factors—including age, gender, qualification (general practitioner or specialist), prior education on stem cells, and engagement with scientific literature—that influence practitioners' awareness, perceptions, and readiness to adopt regenerative approaches in clinical practice. By examining both univariate associations and multivariate predictors of good knowledge, positive attitude, and favorable clinical practice intention, the study sought to elucidate the determinants underlying the observed knowledge–practice gap. The ultimate objective was to generate evidence-based insights that can guide curricular integration, continuing professional development initiatives, and national policy frameworks, thereby supporting the safe, ethical, and effective translation of regenerative dentistry into routine dental practice across the Kingdom of Saudi Arabia.

## 2 Materials and methods

### 2.1 Study design and ethical considerations

This descriptive, cross-sectional, questionnaire-based study was conducted across the Kingdom of Saudi Arabia between November 2024 and August 2025. The study aimed to assess the (KAP) of dental practitioners regarding dental stem cell (DSC) applications and regenerative dentistry. Ethical approval was obtained from the Institutional Review Board (IRB) of [prince Sattam Bin Abdulaziz university] (Approval No. REC-HSD-93-2021), and the research was conducted in accordance with the ethical principles outlined in the Declaration of Helsinki and national ethical standards. All participants were informed about the study objectives and provided electronic informed consent prior to participation.

The study was designed, implemented, and reported in accordance with the Strengthening the Reporting of Observational Studies in Epidemiology (25) guidelines for cross-sectional studies to ensure methodological transparency, reproducibility, and completeness. Additionally, the online survey component adhered to the Checklist for Reporting Results of Internet E-Surveys (26) framework to maintain rigor in participant recruitment, informed consent, and data integrity.

### 2.2 Setting and sample

The target population comprised dental practitioners actively practicing in Saudi Arabia, including general dentists and specialists such as endodontists, orthodontists, oral surgeons, prosthodontists, periodontists, and pedodontists. Participation was voluntary, and anonymity was guaranteed, with no personal identifiers collected or stored. The survey link was distributed to dental practitioners across multiple social media platforms including WhatsApp, X (formerly Twitter), Telegram, Facebook, Snapchat, and Instagram to promote broad participation from dentists across various regions of Saudi Arabia using a self-administered online questionnaire designed on Google Forms. The sample size was determined using the formula for cross-sectional studies with a 95% confidence level, 5% margin of error, and 50% expected prevalence, yielding a minimum required sample of 384 participants. To enhance statistical power and account for potential incomplete responses, the survey remained open until an adequate sample exceeding this threshold was achieved. Only fully completed questionnaires were included in the final analysis. The target population consisted of current general and specialist practitioners in the Kingdom. All participants were informed of the study's objectives, and electronic informed consent was obtained before participation. Inclusion criteria required participants to be actively engaged in clinical practice in Saudi Arabia during the study period. Responses that failed to meet the inclusion criteria were excluded to maintain data quality and reliability.

To ensure data quality and validity, only fully completed and verified responses were included in the final analysis.

### 2.3 Survey instrument development and validation

#### 2.3.1 Initial development

The survey instrument was adapted from validated questionnaires used in previous studies assessing knowledge and attitudes toward regenerative dentistry and stem cell therapy (16, 22).

#### 2.3.2 Expert validation

Content validity was established through a structured expert review involving six dental specialists representing relevant disciplines: Endodontics (*n* = 2), Oral Biology/Regenerative Dentistry (*n* = 2), Dental Public Health/Education (*n* = 1), and Oral Surgery (*n* = 1). This multidisciplinary panel was selected to ensure comprehensive evaluation of the questionnaire's clinical relevance, scientific accuracy, and educational appropriateness for assessing knowledge, attitudes, and clinical practice intentions related to dental stem cell applications.

Each expert independently evaluated all questionnaire items for relevance, clarity, comprehensiveness, and conceptual accuracy using a 4-point ordinal scale (1 = not relevant, 4 = highly relevant). Content validity was assessed following the standardized methodology described by Polit and Beck (2006, 2007) and Boateng et al. (2018) (27–29). The Item-Level Content Validity Index (I-CVI) and the Scale-Level Content Validity Index using the averaging method (S-CVI/Ave) were calculated. Items with an I-CVI ≥ 0.83 were retained, reflecting acceptable agreement among at least five of the six experts. The overall S-CVI/Ave value of 0.92 indicated excellent content validity for the finalized questionnaire.

In addition to expert review, a pilot study involving 20 practicing dentists was conducted to assess face validity, clarity of wording, logical flow, internal coherence, and ease of completion in an online format. Feedback from both expert reviewers and pilot participants resulted in minor revisions only, including clarification of wording, refinement of terminology, and standardization of response options. Based on expert feedback, several questionnaire items were refined. These revisions included clarifying the list of dental stem cell sources (e.g., DPSCs, SHED, SCAP, DFPCs, and PDLSCs), rewording the item addressing stem cell differentiation potential, refining the attitude item comparing regenerative therapies with conventional treatments, and improving the clinical scenario related to pulp regeneration in necrotic immature teeth. No items were removed during validation, but wording adjustments were implemented to enhance clarity and clinical relevance. Following these modifications, the questionnaire was finalized and implemented for data collection in the present study. Internal consistency reliability of the questionnaire domains was assessed using Cronbach's alpha coefficient. The obtained values were *α* = 0.69 for the knowledge domain, *α* = 0.71 for the attitude domain, and *α* = 0.48 for the clinical practice intention domain. The knowledge and attitude domains demonstrated acceptable internal consistency, while the lower value for the practice domain may be attributed to the limited number of items included in that section.

### 2.4 Final instrument

The finalized questionnaire consisted of items structured into four domains that comprehensively assessed dentists' knowledge, attitudes, and clinical practice intentions regarding DSC and regenerative dentistry. The first domain captured participants' demographic and professional characteristics, including age, gender, qualification (general practitioner or specialist), prior education on stem cells, and frequency of reading scientific journals. The second domain evaluated knowledge of DSC through six key items addressing stem cell types, dental tissue sources (such as DPSCs, SHED, SCAP, DFPCs, and PDLSCs), differentiation potential, and clinical applications. Participants who correctly answered at least 60% of the items (≥4 of 6) were classified as having good knowledge.

The third domain assessed attitudes toward dental stem cell–based regenerative dentistry through six key items that captured practitioners' perceptions, motivation, and readiness to engage with regenerative innovations. A positive attitude was defined as correctly responding to ≥60% of attitude items (≥4 of 6). The fourth domain examined clinical practice intention, capturing dentists' self-reported readiness to adopt regenerative endodontic procedures, recognize stem cell–related clinical scenarios (such as root formation or pulp regeneration), and select pulp regeneration as an optimal treatment choice for necrotic immature teeth. A good practice intention was defined as scoring ≥50% on this domain (≥1 of 2).

### 2.5 Data collection procedure

Data collection was carried out electronically using Google Forms, allowing for efficient and secure online participation. The survey link was disseminated through various social media platforms and professional networks to ensure broad national coverage and reach dental practitioners across different regions of Saudi Arabia. To optimize response rates, reminder notifications were issued at two-week intervals throughout the data collection period. Participation was entirely voluntary and anonymous, and no personally identifiable information was requested or stored to maintain confidentiality. The questionnaire was configured with mandatory response fields to prevent submission of incomplete surveys; therefore, no missing data were recorded, and all 493 responses were complete and included in the final analysis. Quality assurance procedures—including timestamp validation and duplicate entry screening—were implemented to ensure accuracy, completeness, and consistency of the final dataset before proceeding to statistical analysis.

### 2.6 Statistical analysis

Descriptive statistics were used to summarize the demographic characteristics of the study population, including age, gender, qualification, frequency of reading scientific journals, and prior education on stem cells. These variables were presented as frequencies and percentages.

Knowledge, attitude, and clinical practice intention toward dental stem cell–based regenerative treatments were also summarized using frequency and percentage distributions. For the knowledge domain, the percentage of correct responses was calculated. For the attitude domain, the percentage of participants demonstrating positive attitudes was reported. For the clinical practice intention domain, the percentage of participants expressing willingness to apply stem cell concepts and treatments in clinical practice was calculated.

Participants were classified as having good knowledge and a positive attitude if they correctly answered at least 60% of the listed items (≥4 out of 6). Participants were classified as having good clinical practice intention if they correctly answered at least 50% of the items (≥1 out of 2). These cut-off points were determined in accordance with cognitive assessment frameworks described by Bloom et al. (1956) and recommended standards for validating cut-off scores in educational and assessment research (30–32).

Associations between dentists' demographic and professional characteristics and their knowledge, attitude, and clinical practice intention toward DSC were assessed using the chi-square test.

A multivariate binary logistic regression analysis was performed to identify predictors and associated factors of good knowledge, positive attitude, and good clinical practice intention among participating dentists. The independent variables included age, gender, qualification, frequency of reading scientific journals, and prior education on stem cells. Odds ratios (ORs) with 95% confidence intervals (CIs) were calculated.

All statistical analyses were conducted using IBM SPSS Statistics for Windows, version 25.0 (IBM Corp., Armonk, NY, USA). Statistical significance was set at *p* < 0.05.

### 2.7 Quality control measures

To ensure data validity, reliability, and consistency with the study objectives, multiple quality control measures were implemented throughout the design, data collection, and analysis phases of this cross-sectional survey. The survey instrument was adapted from previously validated questionnaires and underwent expert review and pilot testing, as described in the instrument development section, to ensure content validity, clarity, and relevance to dental practitioners.

To minimize selection bias, the survey link was disseminated nationwide through professional and social media platforms commonly used by dentists, including WhatsApp, X (formerly Twitter), Telegram, Facebook, Snapchat, and Instagram. Participation was open to actively practicing dental practitioners without restriction by age, gender, or specialty. This recruitment approach yielded 493 fully completed responses, including general dental practitioners (52.3%) and specialists (47.7%), with a broad distribution of age and gender, consistent with the characteristics reported in Table 1.

**Table 1:** Characteristics of dentists included in the study (*n* = 493).

Characteristic	Category	Number (*n*)	Percent (%)
Age (years)			
	≤30	287	58.2%
	31–50	177	35.9%
	>50	29	5.9%
Gender			
	Male	284	57.6%
	Female	209	42.4%
Qualification			
	General dental practitioner	258	52.3%
	Specialist dentist	235	47.7%
Reading scientific journals			
	Weekly	109	22.1%
	Monthly	122	24.7%
	Once in a while	208	42.2%
	Never	54	11.0%
Received education on stem cells			
	Yes	197	40.0%
	No	296	60.0%

To reduce response bias, the questionnaire was neutrally framed as a professional knowledge and clinical practice survey, without highlighting stem cell expertise or anticipated outcomes. Participation was voluntary and anonymous, and no personally identifiable information was collected.

Data cleaning procedures were performed prior to analysis. All responses were screened for completeness and internal consistency. Duplicate submissions were removed by cross-checking IP addresses, and incomplete or inconsistent responses were excluded. Data coding, domain scoring, and statistical outputs were independently verified by two researchers, with discrepancies resolved by consensus.

The study was conducted and reported in accordance with the STROBE guidelines for cross-sectional studies, ensuring methodological transparency and reproducibility. Collectively, these quality control measures strengthened the internal validity and robustness of the findings, particularly in identifying factors—such as prior stem cell education and specialist qualification—associated with higher knowledge levels and more positive attitudes toward dental stem cell–based regenerative practices.

## 3 Results

### 3.1 Characteristics of the study population

A total of 493 dentists participated in the survey. The majority of participants were aged ≤30 years (*n* = 287, 58.2%), while only 29 (5.9%) were older than 50 years. Male dentists outnumbered females (*n* = 284, 57.6% vs. 209, 42.4%).

General dental practitioners constituted a slightly higher proportion of the sample compared with specialist dentists (258, 52.3% vs. 235, 47.7%).

Regarding reading scientific journals, most dentists reported reading journals once in a while (*n* = 208, 42.2%), while 54 (11.0%) reported never reading scientific journals. However, 231 dentists (46.8%) reported regular reading habits, either weekly (22.1%) or monthly (24.7%).

Only 197 dentists (40.0%) reported having attended an educational activity related to stem cells, such as a course, program, or lecture, whereas the majority (60.0%) had not received any formal education on stem cells.

The characteristics of the dentists included in this study are presented in Table 1.

### 3.2 Knowledge, attitude, and clinical practice intention toward dental stem cell–based regenerative treatments

Among the 493 dentists surveyed, only 16.7% (*n* = 82) demonstrated good knowledge of DSC (Table 2). Most were aware of the main dental stem cell sources (76.3%), but fewer correctly identified the specific dental sources (56.6%) or both embryonic and adult stem cell types (21.3%) (Table 2). Awareness of other aspects, such as types of stem cells, their differentiation potential, and applications in dentistry, ranged from 43.2% to 49.1%.

**Table 2:** Knowledge, attitude, and clinical practice intention toward dental stem cell–based regenerative treatments (*n* = 493).

Domain	Item	*n* (%)
Knowledge		
	Aware of different types of stem cells	242 (49.1%)
	Aware of main dental sources (DPSCs, SHED, SCAP, DFPCs, PDLSCs)	376 (76.3%)
	Correctly identify dental sources (pulp, papilla, and gingiva)	279 (56.6%)
	Aware that dental stem cells can develop into non-dental tissues	236 (47.9%)
	Knowledge of applications of stem cells in dentistry	213 (43.2%)
	Correctly identify both embryonic and adult stem cell types	105 (21.3%)
Good knowledge of dental stem cells		82 (16.7%)
Attitude		
	Willing to refer patients for regenerative treatment	356 (72.2%)
	Interested in attending advanced stem cell training	350 (71.0%)
	Believe regenerative technology is applicable in dentistry	327 (66.3%)
	Believe dental stem cell banking is useful	304 (61.7%)
	Perceive regenerative treatment as better than implants	279 (56.6%)
	Likely to recommend regenerative treatment to patients	455 (92.3%)
Positive attitude toward dental stem cell–based regenerative treatment		411 (83.3%)
Practice intention		
	Recognize root formation/pulp regeneration as stem cell-related	304 (61.7%)
	Select pulpal regeneration as an optimal clinical choice	45 (9.1%)
Good clinical practice intention in applying dental stem cell concepts		247 (50.1%)

Participants were classified as having good knowledge and a positive attitude if they correctly answered ≥60% of the listed items (≥4 out of 6), and as having good clinical practice intention if they correctly answered ≥50% of the items (≥1 out of 2).

Attitudes toward dental stem cell–based regenerative treatments were generally positive, with 83.3% (*n* = 411) classified as having a positive attitude. A majority were likely to recommend regenerative treatments (92.3%) and were willing to refer patients (72.2%). Most also recognized the usefulness of dental stem cell banking (61.7%) and believed regenerative technology is applicable in dentistry (66.3%).

Regarding clinical practice intention, only half (50.1%, *n* = 247) showed good intention to apply stem cell concepts (Table 2). While 61.7% recognized root formation or pulp regeneration as stem cell–related, only 9.1% would choose pulpal regeneration as an optimal clinical approach.

The former results are shown in Table 2. Overall, the results suggest that although dentists hold positive attitudes toward dental stem cell therapies, knowledge and readiness to apply these concepts in practice remain limited.

### 3.3 Associations between dentists' characteristics and knowledge, attitude, and practice toward dental stem cells

Chi-square analysis revealed several significant associations between dentists' characteristics and their knowledge, attitude, and practice regarding DSC (Table 3).

**Table 3:** Associations between dentists' characteristics and knowledge, attitude, and practice toward dental stem cells (*n* = 493).

Characteristic	Knowledge (Good/n %)	*p*	Attitude (Positive/n %)	*p*	Practice (Good/n %)	*p*
Age (years)						
≤30 (*n* = 287)	118 (41.1%)	0.686	189 (65.9%)	<0.001*	150 (52.3%)	0.930
31–50 (*n* = 177)	76 (42.9%)		145 (81.9%)		91 (51.4%)	
>50 (*n* = 29)	10 (34.5%)		17 (58.6%)		16 (55.2%)	
Gender						
Male (*n* = 284)	114 (40.1%)	0.577	197 (69.4%)	0.344	148 (52.1%)	1.000
Female (*n* = 209)	90 (43.1%)		154 (73.7%)		109 (52.2%)	
Qualification						
General dental practitioners (*n* = 258)	100 (38.8%)	0.441	161 (62.4%)	0.006*	133 (51.6%)	0.573
Specialist dentist (*n* = 235)	104 (44.3%)		190 (80.9%)		124 (52.8%)	
Scientific journal reading						
Regular (Weekly/Monthly) (*n* = 231)	103 (44.6%)	0.205	181 (78.4%)	0.001*	132 (57.1%)	0.045*
Occasional/Never (*n* = 262)	101 (38.5%)		170 (64.9%)		125 (47.7%)	
Prior stem cells education						
Yes (*n* = 197)	102 (51.8%)	<0.001*	160 (81.2%)	<0.001*	111 (56.3%)	0.151
No (*n* = 296)	102 (34.5%)		191 (64.5%)		146 (49.3%)	

* Denotes significant difference at *p* < 0.05 as indicated by Chi-square statistics.

#### 3.3.1 Knowledge

Good knowledge of DSC was significantly associated with prior stem cell education, with 51.8% of dentists who had received education demonstrating good knowledge compared to 34.5% of those without prior education (*p* < 0.001). No significant association was observed between knowledge and age, gender, qualification, or frequency of reading scientific journals.

#### 3.3.2 Attitude

Positive attitude toward dental stem cell–based regenerative treatments was significantly associated with age, qualification, journal reading, and prior education. Dentists aged 31–50 years showed the highest proportion of positive attitudes (81.9%, Table 2), while those above 50 years showed the lowest (58.6%) (*p* < 0.001). Specialist dentists had a higher rate of positive attitudes compared to general dental practitioners (80.9% vs. 62.4%, *p* = 0.006). Regular readers of scientific journals were more likely to have a positive attitude (78.4% vs. 64.9%, *p* = 0.001), as were those with prior stem cell education (81.2% vs. 64.5%, *p* < 0.001).

#### 3.3.3 Practice intention

Good clinical practice intention was significantly associated with regular reading of scientific journals, with 57.1% of regular readers showing good practice intention compared to 47.7% of occasional or non-readers (*p* = 0.045).

Overall, prior education on stem cells and engagement with scientific literature were key factors associated with better knowledge, more positive attitudes, and greater clinical practice intention regarding dental stem cell–based regenerative treatments.

### 3.4 Predictors of good knowledge, positive attitude, and good practice

Multivariate binary logistic regression was performed to identify independent predictors of good knowledge, positive attitude, and good practice regarding DSC among participating dentists (Table 4).

**Table 4:** Predictors and associated factors of good knowledge, positive attitude, and good practice among participating dentists.

Predictor/Associated Factor	Knowledge (Good) OR (95% CI)	*P*	Attitude (Positive) OR (95% CI)	*p*	Practice (Good) OR (95% CI)	*p*
Age (years)						
≤30	[Reference]		[Reference]		[Reference]	
31–50	0.97 (0.66–1.44)	0.887	2.03 (1.27–3.24)	0.003*	0.88 (0.60–1.30)	0.525
>50	0.62 (0.27–1.41)	0.258	0.55 (0.25–1.24)	0.152	1.01 (0.46–2.20)	0.975
Gender						
Male	[Reference]		[Reference]		[Reference]	
Female	1.22 (0.84–1.77)	0.288	1.33 (0.88–2.02)	0.174	1.01 (0.70–1.45)	0.967
Qualification						
General practitioner	[Reference]		[Reference]		[Reference]	
Specialist	1.23 (0.74–2.03)	0.441	2.03 (1.23–3.36)	0.006*	1.08 (0.70–1.66)	0.573
Scientific journal reading						
Occasional/Never	[Reference]		[Reference]		[Reference]	
Regular (Weekly/Monthly)	0.99 (0.67–1.48)	0.969	1.41 (0.90–2.19)	0.132	1.40 (0.95–2.07)	0.086
Prior stem cells education						
No	[Reference]		[Reference]		[Reference]	
Yes	2.13 (1.43–3.19)	<0.001*	2.10 (1.31–3.36)	0.002*	1.19 (0.80–1.76)	0.389

The odds ratio and 95% confidence interval were calculated by a multivariate binary logistic model.

* Significance in this analysis was defined at *p* < 0.05.

#### 3.4.1 Knowledge

Prior stem cell education was the only significant predictor of good knowledge. Dentists who had received prior education were more than twice as likely to demonstrate good knowledge compared to those without education (OR=2.13, 95% CI: 1.43–3.19, *p* < 0.001). Age, gender, qualification, and frequency of reading scientific journals were not significantly associated with knowledge.

#### 3.4.2 Attitude

A positive attitude was independently associated with age, qualification, and prior stem cell education. Dentists aged 31–50 years were twice as likely to have a positive attitude compared to those ≤30 years (OR=2.03, 95% CI: 1.27–3.24, *p* = 0.003). Specialist dentists were also more likely to have a positive attitude than general practitioners (OR=2.03, 95% CI: 1.23–3.36, *p* = 0.006). Prior stem cell education was a significant predictor of positive attitude, with educated dentists being twice as likely to hold a positive attitude (OR=2.10, 95% CI: 1.31–3.36, *p* = 0.002). Gender and journal reading were not significant predictors.

#### 3.4.3 Practice intention

No significant predictors of good clinical practice intention were identified in the multivariate model. Although regular journal readers and dentists with prior education had higher odds of good practice intention, these associations did not reach statistical significance.

In summary, prior stem cell education consistently predicted both good knowledge and positive attitudes, while specialist dentists were also more likely to have a positive attitude compared to general practitioners. Clinical practice intention, however, appeared independent of the examined demographic and professional factors.

## 4 Discussion

This cross-sectional study offers an in-depth evaluation of Saudi dentists' knowledge, attitudes, and clinical practice intentions (KAP) toward (DSC)–based regenerative therapies. With a large and statistically adequate sample (*n* = 493), surpassing the minimum threshold for robust inferential analysis, this study provides a reliable reflection of the professional landscape in Saudi Arabia. The high response rate and representative sampling strengthen its validity and generalizability, allowing confident identification of educational and structural determinants influencing regenerative practice adoption.

### 4.1 Knowledge and awareness

Only 16.7% of the surveyed dentists demonstrated good knowledge of DSC (Table 2). Although a majority were aware of the main dental stem cell sources—such as dental pulp stem cells (DPSC), stem cells from human exfoliated deciduous teeth (SHED), stem cells from the apical papilla (SCAP), dental follicle progenitor cells (DFPCs), and periodontal ligament stem cells (PDLSCs) (76.3%)—substantial knowledge gaps were evident. Just over half correctly identified specific dental tissue sources (56.6%), while fewer participants were aware that DSC can differentiate into non-dental tissues (47.9%) or demonstrated knowledge of their clinical applications in dentistry (43.2%). Notably, only 21.3% correctly identified both embryonic and adult stem cell types, highlighting limited understanding of fundamental stem cell biology (Table 2). These findings are consistent with previous regional and international research highlighting widespread conceptual gaps in stem cell biology among dental practitioners (33, 34). Similarly, Desai et al. (35) reported that insufficient curricular exposure and limited professional training were key barriers to comprehensive understanding.

Prior education on stem cells was the strongest determinant of good knowledge (OR=2.13, *p* < 0.001, Table 3, 4). This reinforces evidence from Goswami M et al. and Yamada S et al. (34, 36), both of whom found that structured teaching and continuing education significantly improved comprehension of regenerative concepts. This correlation underscores the importance of embedding formal regenerative medicine modules within dental curricula and expanding postgraduate professional development opportunities.

### 4.2 Attitudes toward regenerative dentistry

Encouragingly, most respondents (83.3%, Table 2) demonstrated a positive attitude toward dental stem cell–based regenerative therapies. This aligns with previous findings in Saudi Arabia (37, 38) and beyond, suggesting growing enthusiasm toward biological approaches to dental care. Mid-career dentists (*p* = 0.003, Table 4) and specialists (*p* = 0.006, Table 4) demonstrated significantly greater optimism toward dental stem cell–based regenerative treatments, likely reflecting their broader exposure to advanced restorative concepts and clinical innovations. Additionally, prior education on stem cells was a strong predictor of positive attitude (*p* = 0.002, Table 4) underscoring the role of structured academic exposure in shaping professional perspectives. However, as emphasized by Yamada S et al. (36), such optimism frequently does not translate into confident clinical readiness. Persistent barriers—including limited training infrastructure, ethical uncertainties, and regulatory ambiguity—continue to impede practical implementation. Furthermore, Alomar RK et al. (39) proposed that developing coordinated national initiatives, such as dental stem cell banking programs and centralized research collaborations, could enhance professional trust and facilitate the clinical integration of regenerative dentistry.

### 4.3 Clinical practice intention

Despite favourable perceptions, only 50.1**%** (Table 2) of respondents expressed readiness to adopt DSC based therapies in practice. This “knowledge–practice gap” is well-documented in literature, reflecting the lag between conceptual awareness and clinical application (35, 40).

Although most demographic factors did not significantly influence clinical practice intention (*p* > 0.05), dentists who regularly engaged with scientific literature demonstrated a markedly higher likelihood of good practice behavior (57.1% vs. 47.7%; *p* = 0.045, Table 3), underscoring the importance of continuous academic engagement. According to Al-Huthaifi et al. (2025, preprint) (41), institutional shortcomings, such as limited laboratory access, weak interdisciplinary coordination, and the absence of standardized clinical protocols, remain key barriers hindering the clinical adoption of dental stem cell–based therapies despite theoretical support. Encouragingly, Bucchi et al. (42) reported that frequent participation in continuing education and sustained interaction with scientific research significantly enhance dentists' confidence and readiness to apply regenerative procedures. Collectively, these findings highlight the need for structured, evidence-based professional development initiatives that prioritize hands-on training and regulatory competence to bridge the gap between knowledge and clinical implementation.

### 4.4 Educational and policy implications

Despite strong enthusiasm for dental stem cell–based therapies among Saudi dentists, this study identifies a clear gap between positive attitudes and clinical preparedness. Limited knowledge and only moderate practice intention indicate that education, rather than motivation, is the principal barrier to adoption. Integrating regenerative biology, stem cell applications, and tissue engineering into undergraduate curricula—supported by experiential learning such as simulation-based training and supervised research—may help translate interest into competency, consistent with the observed impact of prior stem cell education (43).

At the policy level, the lack of independent predictors for clinical practice intention suggests system-level barriers to implementation. Establishing national frameworks for ethical stem cell sourcing, banking, and clinical application, alongside standardized accreditation and clinical protocols, would support safe and confident adoption. Embedding regenerative dentistry within national education and continuing professional development policies will promote sustainable integration and align clinical practice with Saudi Vision 2030 goals (44).

### 4.5 Limitations

Despite its strong national representation and robust analytical framework, this study has several limitations. As a cross-sectional survey, causal relationships between prior education, attitudes, and clinical practices cannot be firmly established. The associations observed indicate correlation rather than causation. Second, the study relied on self-reported responses, which are inherently vulnerable to recall and social desirability bias, participants may have overestimated their knowledge or readiness to adopt regenerative therapies. In addition, multiple bivariate comparisons were performed when examining associations between dentists' characteristics and KAP outcomes. Although these analyses were exploratory, performing multiple statistical tests may increase the risk of Type I error. Therefore, borderline significant findings should be interpreted with caution.

Third, the online distribution method may have introduced selection bias by favoring digitally active or academically inclined dentists, potentially limiting the representation of less-connected practitioners. Additionally, while the survey instrument was validated by experts and pilot-tested, variation in interpretation of certain knowledge and attitude items could not be eliminated. Furthermore, the clinical practice intention domain included only two items, which may limit the precision of the classification and reduce differentiation between respondents. Finally, contextual variables such as institutional support, resource availability, or hands-on exposure were not assessed, which may have influenced practical readiness and adoption trends.

To advance the integration of regenerative dentistry in Saudi Arabia, longitudinal and interventional studies should be conducted to evaluate how targeted educational reforms and structured continuing professional development programs influence clinical competency over time. Establishing national training centers, simulation-based modules, and collaborative research frameworks can provide sustainable pathways for experiential learning. Future research should also explore institutional, ethical, and infrastructural determinants including access to laboratory facilities, ethical compliance systems, and patient awareness to develop comprehensive strategies that promote the safe and effective translation of regenerative science into everyday dental practice.

## 5 Conclusion

Saudi dentists show strong interest in regenerative dentistry but limited knowledge and clinical readiness. While most hold positive attitudes (83.3%), only a small proportion demonstrate sufficient understanding (16.7%) or practical readiness (50.1%). Prior stem cell education is the key factor influencing both knowledge and attitude. Bridging this gap requires integrating regenerative concepts into dental curricula, expanding professional training, and establishing ethical and regulatory frameworks. Strengthening education and governance will enable Saudi Arabia to translate regenerative dentistry from theory into clinical reality, supporting the nation's Vision 2030 healthcare goals.

## Data Availability

The raw data supporting the conclusions of this article will be made available by the authors, without undue reservation.
